# Use of BacterioScan 216Dx to reduce antibiotic use for suspected urinary tract infection in the emergency department

**DOI:** 10.1017/ash.2025.10238

**Published:** 2025-11-27

**Authors:** Kelly Gao, Vishak Kumar, Giovanni Divinagracia, Thomas J. Kirn, Bert Berla, Keith S. Kaye, Ahmed Abdul Azim

**Affiliations:** 1 Icahn School of Medicine, New York, NY, USA; 2 Division of Allergy, Immunology and Infectious Diseases, Rutgers Robert Wood Johnson Medical School, New Brunswick, NJ, USA; 3 Department of Pathology and Laboratory Medicine, Robert Wood Johnson Medical School, New Brunswick, NJ, USA; 4 Bacterioscan Inc, St Louis, MO, USA

## Abstract

We assessed whether negative BacterioScan urine screen results could reduce unnecessary antibiotic use in emergency department patients with positive urinalyses. Among 82 patients with concordant negative results for both BacterioScan and urine culture, 10% were prescribed antibiotics. Real-time access to BacterioScan may have led to antibiotic avoidance in these patients.

## Background

Inappropriate antibiotic use has led to antimicrobial resistance, a growing global public health concern.^1^ Urinary tract infections (UTI) are over- and misdiagnosed in hospitals, especially in acute care settings.^2,3^ Twenty to 40% of suspected UTIs yield negative culture results and therefore are in most cases, by definition, not UTIs; thus, antibiotic treatment in these instances is typically inappropriate.^4^ However, emergency department (ED) patients are often empirically treated with and discharged on antibiotics before urine culture (UC) results become available.^1^

BacterioScan 216Dx (BacterioScan, Inc.) is a Food and Drug Administration-cleared device that allows for point-of-care analysis of urine samples for many pathogenic bacteria in three hours, as opposed to conventional UCs, for which results can take up to 48 h to become available.^5^ The BacterioScan system uses the trend of optical density of a urine sample mixed with bacterial growth media to identify urine samples containing ≥50,000 colony-forming units (CFU)/ml of a uropathogen.^5^

Our study examines the potential for negative BacterioScan results to guide antibiotic decision-making in the ED and ED observation unit prior to the availability of UC results.

## Methods

We performed a retrospective chart review at Robert Wood Johnson University Hospital (RWJUH), a tertiary academic medical center with a mixed urban-suburban patient population. Patients who visited the ED from December 1, 2023, to April 8, 2024, with a positive urinalysis (UA) that reflexed to UC were included in the study. We included both clean-catch urine and urinary catheter samples. Positive UA was defined as white blood cells (WBC) > 5 cells/high power field (hpf), leukocyte esterase positive, or nitrite positive. Positive UC was defined as growth of ≥10^3^ CFU/ml of a potential pathogen. A research technician identified ED urine samples sent to the laboratory that met the inclusion criteria. These urine samples were tested using BacterioScan (typically within 24 h after having arrived in the microbiology laboratory), but results were not released to treating clinicians. BacterioScan results were only used for retrospective analysis.

All urine testing with BacterioScan was conducted according to the manufacturer’s instructions. In brief, 2.5 ml of tryptic soy broth (TSB) was dispensed to each multicuvette by manual pipetting. Next, 360 μl of each urine specimen was individually dispensed to a multicuvette and mixed by manual pipetting up and down 4 to 5 times. After the lids were closed, sample information was entered into the 216Dx graphical user interface (GUI). Following a 3-hour incubation and evaluation of all loaded samples, the GUI returned a qualitative “presumptive positive” or “negative” result.

Patient demographics (age, sex, race and ethnicity) were recorded along with BacterioScan and UC results. Antibiotic-related variables included indication for treatment, empiric antibiotic choice, route of administration, and duration of treatment. Microbiology data included uropathogen identification without susceptibility results.

Patients admitted to the hospital were excluded from the study as the objective was to examine BacterioScan’s use in the ED and ED observation unit settings for patients being discharged, where treatment decisions are more time sensitive.

## Results

During the study period, 600 patients in the ED with a positive UA that was reflexed to UC were included. BacterioScan was negative in 133 of the 600 patients. Thirty-four of these patients were admitted to the hospital and were thus excluded. The study cohort included 99 patients who did not get admitted to the hospital. These 99 patients ranged from 16 to 90 years of age with a mean age of 47.67 (SD 20.67); 66% were female; 36% identified as White, 16% African American, 4% Asian, and 44% were categorized as other (eg, they often preferred not to respond). Thirty-three percent of patients identified as Hispanic and 67% as non-Hispanic.

In most cases, negative BacterioScan results were concordant with negative UC results (82/99). Of these 82 concordant patient specimens, 8 (9.75%) were treated empirically with antibiotics for suspected UTI for a median duration of 5.4 days (range: 1–10 d). The characteristics of empiric antibiotic choices and durations are described in Table 1. Five of the 8 patients (62.5%) were discharged with antibiotics before culture results were available. These five patients were treated with at least 3 days of antibiotics. In the other 3 of 8 cases, physicians were able to stop treatment after culture results became available.

**Table 1.** BacterioScan negative and urine culture negative patients treated for urinary tract infection

Patient	Antibiotic used	Route	Duration (days)
1	β-lactam/β-lactamase inhibitor, third generation cephalosporin	IV	3
2	β-lactam/β-lactamase inhibitor, Fluoroquinolone	IV, PO	8
3	Third generation cephalosporin, Fluoroquinolone	IV, PO	9
4	Fourth generation cephalosporin	IV	1
5	Fourth generation cephalosporin	IV	1
6	First generation cephalosporin	PO	10
7	Tetracycline	PO	10
8	Nitrofurantoin	PO	1

Seventeen of 99 patients had discordant results (ie, were BacterioScan negative and UC positive) (Table 2). Sixteen (94%) of these patients had UC with <50,000 CFU/ml, which is below the limit for BacterioScan’s urine pathogen detection. Thirteen patients (76%) had growth of mixed bacterial flora in the urine. One of the 17 patients (6%) had a UC positive for a uropathogen, *Streptococcus agalactiae,* at ≥100,000 CFU/ml; the patient had abdominal pain secondary to menses but was deemed and ultimately diagnosed by treating clinicians as having asymptomatic bacteriuria not requiring treatment.

**Table 2.** Uropathogen identified for bacterioScan negative and urine culture positive patients

Patient	Urine pathogen	Colony forming units
1	*Klebsiella pneumoniae*	<50,000
2	*Streptococcus agalactiae*	<50,000
3	*Streptococcus agalactiae* *	≥100,000
4	*Proteus mirabilis*	<50,000
5	Mixed gram-positive flora	<50,000
6	Mixed gram-positive flora	<50,000
7	Mixed gram-positive flora	<50,000
8	Mixed gram-positive flora	<50,000
9	Mixed gram-positive flora	<50,000
10	Mixed gram-positive flora	<50,000
11	Mixed gram-positive flora	<50,000
12	Mixed gram-positive flora	<50,000
13	Mixed gram-positive flora	<50,000
14	Mixed gram-positive flora	<50,000
15	Mixed gram-positive flora	<50,000
16	Gram-negative bacilli	<50,000
17	Mixed gram-negative and gram-positive organisms	<50,000

* One of the 17 patients (6%) had a UC positive for a uropathogen, *Streptococcus agalactiae,* at ≥100,000 CFU/ml; the patient had abdominal pain secondary to menses but was deemed and ultimately diagnosed by treating clinicians as having asymptomatic bacteriuria not requiring treatment.

## Discussion

Approximately 10% of patients with UA positive, BacterioScan negative, and UC negative results who visited RWJUH between December 2023 and April 2024 could have avoided antibiotic treatment had BacterioScan results been available to clinicians in real time. The median reduction of antibiotic days that could have been avoided in these patients was greater than 5 days.

Our study expands upon the current literature of antibiotic prescription for suspected UTIs in the ED. A prior study in 2015 of ED patients in an academic urban center in Ohio demonstrated that 20% of ED patients were prescribed antibiotics despite ultimately having negative UCs [2].

One limitation in our study was that Bacterioscan testing was performed only on UA positive specimens, which is a different population than is reflected in studies described as part of the instrument’s validation, where urine samples were tested with Bacterioscan regardless of UA results.^5^ If UA negative specimens had been included, a negative BacterioScan result likely would have been an even stronger predictor of a negative UC. In addition, data were collected from a single academic tertiary center. Another limitation is that the three-hour test turnaround time is based on “on instrument” time, which means that in a busy ED, turnaround time will likely be longer.

Our study highlights how inappropriate prescription of empiric antibiotics in acute care settings for suspected UTIs remains an ongoing public health concern. Because traditional UCs can take up to 48 hours to finalize, ED patients with suspected UTIs are often discharged with antibiotics before culture results are known, based on a UA result showing the presence of pyuria, leukocyte esterase, or nitrites. Unfortunately, clinical experience and published data reveal that the presence of pyuria on a UA can trigger antibiotic prescribing regardless of patients’ asymptomatic status, in part because UCs are not available in a timely manner.^3^ BacterioScan can be particularly useful for emergency room patients in point-of-care analysis, providing rapid results compared to conventional UCs. Given the ongoing need and opportunities for improvements in antibiotic stewardship processes in the ED and for patients being evaluated for potential UTI, BacterioScan can help reduce unnecessary antibiotic prescriptions. Our findings provide important context for understanding the potential impact of BacterioScan in urgent care settings in the setting of positive UA findings.
